# Zinc as a complementary treatment for cancer patients: a systematic review

**DOI:** 10.1007/s10238-020-00677-6

**Published:** 2021-01-26

**Authors:** C. Hoppe, S. Kutschan, J. Dörfler, J. Büntzel, J. Büntzel, Jutta Huebner

**Affiliations:** 1 Klinik für Innere Medizin II, Hämatologie und Internistische Onkologie, Universitätsklinikum Jena, Am Klinikum 1, 07747 Jena, Germany; 2 Klinik für HNO-Erkrankungen, Kopf-Hals-Chirurgie, Südharz-Klinikum Nordhausen, Dr.-Robert-Koch-Str. 39, 99734 Nordhausen, Germany; 3 Klinik für Hämatologie und Medizinische Onkologie, Universitätsmedizin Göttingen, Robert-Koch-Str. 40, 37075 Göttingen, Germany

**Keywords:** Zinc, Cancer, Complementary medicine, Nutritional supplements, Immune system, Side effects

## Abstract

Zinc is a trace element that plays an important role in the immune system and cell growth. The role of zinc in cancer treatment has been discussed for some time, however without reaching an evidenced-based consensus. Therefore, we aim to critically examine and review existing evidence on the role of zinc during cancer treatment. In January 2019, a systematic search was conducted searching five electronic databases (Embase, Cochrane, PsychINFO, CINAHL and PubMed) to find studies concerning the use, effectiveness and potential harm of zinc therapy on cancer patients. Out of initial 5244 search results, 19 publications concerning 23 studies with 1230 patients were included in this systematic review. The patients treated with zinc were mainly diagnosed with head and neck cancer and underwent chemo-, radio- or concurrent radio-chemotherapy. Interventions included the intake of different amounts of zinc supplements and oral zinc rinses. Outcomes (primary endpoints) investigated were mucositis, xerostomia, dysgeusia, pain, weight, dermatitis and oral intake of nutrients. Secondary endpoints were survival data, quality of life assessments and aspects of fatigue, immune responses and toxicities of zinc. The studies were of moderate quality reporting heterogeneous results. Studies have shown a positive impact on the mucositis after radiotherapy. No protection was seen against mucositis after chemotherapy. There was a trend to reduced loss of taste, less dry mouth and oral pain after zinc substitution. No impact was seen on weight, QoL measurements, fatigue, and survival. The risk of side effects from zinc appears to be relatively small. Zinc could be useful in the prevention of oral toxicities during irradiation. It does not help in chemotherapy-induced side effects.

## Introduction

On average, half of all cancer patients in Germany use complementary or alternative medicine (CAM). While mistletoe preparations and homeopathy are commonly used (15% and 6% respectively), most of the patient (35%) use vitamins and trace elements [1, 2]. The most common reasons of patients for using complementary medicine are to strengthen the immune system, to strengthen oneself or to do something for themselves. A survey among breast cancer patients showed, that 80% of patients questioned use CAM to reduce side effects and to boost the immune system [3]. The intake of trace elements is the most popular method of complementary medicine among cancer patients [4]. One of the most often used trace elements is zinc. Despite the heterogeneity of the data, studies on healthy people regarding zinc have shown a trend that it might reduce the duration of a common cold [5]. A meta-analysis revealed beneficial effects in pneumonia [6]. With respect to cancer, several in vitro and in vivo studies point to beneficial effects via the immune system by activating macrophages. Zinc modulates oxidative stress and might help to prevent cancer [7]. In case reports, an improvement of the effects of chemotherapy has been described [8].

CAM is also used under the premise to fight cancer. In this context, some proponents of zinc supplementation for cancer patients point to data showing that a decreased serum concentration of zinc in some types of solid cancers (head and neck, prostate, hepatocellular and pancreatic) seems to be a common event [9, 10].

This review intends to investigate clinical studies on the influence of zinc on cancer therapy-related side effects. It also examines secondary effects on survival, quality of life and the immune system. In addition, there is an examination of the dose used when zinc is administered and any with zinc associated side effects.

## Methods

### Criteria for including and excluding studies in the review

Inclusion and exclusion criteria are listed in Table 1 based on a PICO model. Generally, all original studies with a randomized controlled design or systematic reviews, which cover studies with a randomized controlled design, were included, if they reported patient-relevant outcomes (symptoms, toxicities) after treatment of adult cancer patients with any oral or intravenous intervention containing zinc. All cancer entities were included because of the wide range of application fields. Criteria for rejecting studies were primary prevention, grey literature, other publication types than primary investigation/report (e.g. comments, letters, abstracts), other study types (one-armed/non-controlled studies, case report or series) and study population with only precancerous conditions. Also, studies with more than 20% children (under the age of 18) or if results of adult patients with cancer were not reported separately. Additionally, studies were excluded if they reported no patient centered outcomes (e. g. laboratory parameters). Language restrictions were set to English and German.

**Table 1:** Inclusion and exclusion criteria

PICO	Inclusion criteria	Exclusion criteria
Patient	Cancer patients (all entities and stages) Adult patients (age > 18)	Patients with only precancerous conditions or Carcinoma in situ Preclinical studies Study population with more than 20% children or precancerous conditions
Intervention	Every intervention with zinc (orally or IV)	
Comparison	All possible control groups (placebo, standard care, observation)	Other study types (one-armed/non-controlled studies, case report or series)
Outcome	Primary endpoints were all patient-relevant symptoms/toxicities, secondary endpoints were response data, survival data, and quality of life	No patient- centred data, for example laboratory parameters
Others	Meta-analyses, systematic reviews and RCTs Language: German and English Full publication	Grey literature (conference articles, abstracts, letters, ongoing studies, unpublished literature…)

### Study selection

A systematic research was conducted using five databases (PubMed (Ovid), CINAHL (EBSCO), EMBASE (Ovid), Cochrane CENTRAL and PsychINFO (EBSCO)) in January 2019. For each of these databases, a complex search strategy was developed consisting of a combination of Mesh terms, keywords and text words in different spellings connected to *cancer* and *zinc* (Table 2). After importing the search results into EndNote X6, all duplicates were removed, and a title–abstract screening was carried out by two independent reviewers (CH and JH). In case of disagreement, consensus was reached discussion. Afterwards, all full texts were retrieved and screened again independently by the reviewers. When title and abstract did not have sufficient information for screening purposes, a full text copy was retrieved as well. Additionally, bibliography lists of all retrieved articles were searched for relevant studies.

**Table 2:** Search Strategy

Database	Search strategy
OVID medline	1 zinc/or zinc isotopes/or zinc.mp. or zink.mp. or zn.mp. 2 exp neoplasms/or neoplasm$.mp or cancer$.mp. or tumo?r$.mp. or malignan$.mp. or oncolog$.mp. or carcinom$.mp. or leuk?emia.mp. or lymphom$.mp. or sarcom$.mp. 3 1 AND 2 4 limit 3 to english or limit 3 to german 5 limit 4 to yr = "1995 -Current" 6 (5 and humans/) or (5 not animals/) 7 ((((comprehensive* or integrative or systematic*) adj3 (bibliographic* or review* or literature)) or (meta-analy* or metaanaly* or "research synthesis" or ((information or data) adj3 synthesis) or (data adj2 extract*))).ti,ab. or (cinahl or (cochrane adj3 trial*) or embase or medline or psyclit or (psycinfo not "psycinfo database") or pubmed or scopus or "sociological abstracts" or "web of science" or central).ab. or ("cochrane database of systematic reviews" or evidence report technology assessment or evidence report technology assessment summary).jn. or Evidence Report: Technology Assessment*.jn. or (network adj1 analy*).ti,ab.) or (((review adj5 (rationale or evidence)).ti,ab. and review.pt.) or meta-analysis as topic/or Meta-Analysis.pt.) 8 Randomized controlled trial.pt. or controlled clinical trial.pt. or randomi?ed.ti,ab.or placebo.ti,ab. or drug therapy.sh. or randomly.ti,ab. or trial?.ti,ab. or group?.ti,ab. 9 6 AND (7 OR 8)
OVID Embase	1 exp zinc/or Zinc.mp. or Zink.mp. or Zn.mp. 2 exp neoplasms/or neoplasm$.mp or cancer$.mp. or tumo?r$.mp. or malignan$.mp. or oncolog$.mp. or carcinom$.mp. or leuk?emia.mp. or lymphom$.mp. or sarcom$.mp. 3 1 AND 2 4 limit 3 to english or limit 3 to german 5 limit 4 to yr = "1995 -Current" 6 (5 and humans/) or (5 not animals/) 7 ((((comprehensive* or integrative or systematic*) adj3 (bibliographic* or review* or literature)) or (meta-analy* or metaanaly* or "research synthesis" or ((information or data) adj3 synthesis) or (data adj2 extract*))).ti,ab. or (cinahl or (cochrane adj3 trial*) or embase or medline or psyclit or (psycinfo not "psycinfo database") or pubmed or scopus or "sociological abstracts" or "web of science" or central).ab. or ("cochrane database of systematic reviews" or evidence report technology assessment or evidence report technology assessment summary).jn. or Evidence Report: Technology Assessment*.jn. or (network adj1 analy*).ti,ab.) or (exp Meta-Analysis/or ((data extraction.ab. or selection criteria.ab.) and review.pt.)) 8 crossover procedure/or double blind procedure/or randomized controlled trial/or single blind procedure/or (random$ or factorial$ or crossover$ or (cross adj1 over$) or placebo$ or (doubl$ adj1 blind$) or (singl$ adj1 blind$) or assign$ or allocat$ or volunteer$).ti,ab,de. 9 6 AND (7 OR 8)
Cochrane	#1 [mh zinc] or [mh zinc compounds] or [mh zinc sulphate] or [mh zinc acetate] or zinc or zink or zn #2 [mh neoplasms] or neoplasm* or cancer? or tum*r? or malignan* or oncolog* or carcinom* or leuk*mia or lymphoma? or sarcoma? #3 1 AND 2
EBSCO PsychINFO	S1 DE zinc or TX (zinc or zink or zn) S2 ((DE "Neoplasms" OR DE "Benign Neoplasms" OR DE "Breast Neoplasms" OR DE "Endocrine Neoplasms" OR DE "Leukemias" OR DE "Melanoma" OR DE "Metastasis" OR DE "Nervous System Neoplasms" OR DE "Terminal Cancer") OR (TX neoplasm* OR TX cancer OR TX tumo#r OR TX malignan* OR DE „oncology “ OR TX oncolog* OR TX carcinom* OR TX leuk#emia OR TX lymphoma OR TX sarcoma)) S3 (LA German OR LA English) S4 S1 AND S2 AND S3 S5 ((comprehensive* OR integrative OR systematic*) N3 (bibliographic* OR review* OR literature)) OR (meta-analy* or metaanaly* or "research synthesis" OR ((information OR data) N3 synthesis) OR (data N2 extract*)) OR ((review N5 (rationale OR evidence)) AND DE "Literature Review") OR (AB(cinahl OR (cochrane N3 trial*) OR embase OR medline OR psyclit OR pubmed OR scopus OR "sociological abstracts" OR "web of science" OR central)) OR DE "Meta Analysis" OR (network N1 analy*) S6 DE "Treatment Effectiveness Evaluation" OR DE "Treatment Outcomes" OR DE "Psychotherapeutic Outcomes" OR DE "Placebo" or DE "Followup Studies" OR placebo* OR random* OR "comparative stud*" OR (clinical N3 trial*) OR (research N3 design) OR (evaluat* N3 stud*) OR (prospectiv* N3 stud*) OR ((singl* OR doubl* OR trebl* OR tripl*) N3 (blind* OR mask*) S7 S4 AND (S5 OR S6)
EBSCO Cinahl	S1 MH zinc or TX (zinc or zink or zn) S2 (MH "Neoplasms + " OR TX neoplasm* OR TX cancer OR TX tumo#r OR TX malignan* OR TX oncolog* OR TX carcinom* OR TX leuk#emia OR TX lymphoma OR TX sarcoma OR MH "Precancerous Conditions + " OR TX precancer* OR TX preneoplas*) S3 (LA German OR LA English) S4 S1 AND S2 AND S3 S5 (TI (systematic* n3 review*)) or (AB (systematic* n3 review*)) or (TI (systematic* n3 bibliographic*)) or (AB (systematic* n3 bibliographic*)) or (TI (systematic* n3 literature)) or (AB (systematic* n3 literature)) or (TI (comprehensive* n3 literature)) or (AB (comprehensive* n3 literature)) or (TI (comprehensive* n3 bibliographic*)) or (AB (comprehensive* n3 bibliographic*)) or (TI (integrative n3 review)) or (AB (integrative n3 review)) or (JN “Cochrane Database of Systematic Reviews”) or (TI (information n2 synthesis)) or (TI (data n2 synthesis)) or (AB (information n2 synthesis)) or (AB (data n2 synthesis)) or (TI (data n2 extract*)) or (AB (data n2 extract*)) or (TI (medline or pubmed or psyclit or cinahl or (psycinfo not “psycinfo database”) or “web of science” or scopus or embase)) or (AB (medline or pubmed or psyclit or cinahl or (psycinfo not “psycinfo database”) or “web of science” or scopus or embase or central)) or (MH “Systematic Review”) or (MH “Meta Analysis”) or (TI (meta-analy* or metaanaly*)) or (AB (meta-analy* or metaanaly*)) or TI (network analy*) or AB (network analy*) S6 (MH "Clinical Trials + ") or PT Clinical trial or TX clinic* n1 trial* or TX ((singl* n1 blind*) or (singl* n1 mask*)) or TX ((doubl* n1 blind*) or (doubl* n1 mask*)) or TX ((tripl* n1 blind*) or (tripl* n1 mask*)) or TX ((trebl* n1 blind*) or (trebl* n1 mask*)) or TX randomi* control* trial* or (MH "Random Assignment") or TX random* allocat* or TX placebo* or MH "Placebos") or MH "Quantitative Studies") or TX allocat* random* S7 S4 AND (S5 OR S6)

### Assessment of risk of bias and methodological quality

All characteristics were assessed by two independent reviewers (CH and SK). In case of disagreement, a third reviewer was consulted (JH), and consensus was made by discussion.

#### Risk of bias

The risk of bias in the included studies was analysed with the SIGN checklist for controlled trials Version 2.0 [11] and the AMSTAR-2 instrument for systematic reviews or meta-analyses [12]. In addition, blinding of researchers, blinding of outcome assessment and comparability of groups before treatment not only in terms of demographic variables, but also concerning the outcomes were examined.

#### Methodological quality

The included studies were rated according to the Oxford criteria [13]. Additional criteria concerning methodology were size of population, application of power analysis, dealing with missing data and drop out (report of drop-out reasons, application of intention to treat analysis), adequacy of statistical tests (e.g. control of premises or multiple testing) and selective outcome reporting (report of all assessed outcomes with specification of statistical data as the *p *value).

### Data extraction

Data extraction was performed by one reviewer (CH) and controlled by two independent reviewers (SK/JD, JH). The evidence tables from the national Guideline on Complementary and Alternative Medicine in Oncological Patients of the German Guideline Program in Oncology [14] were used as a template for data extraction.

Concerning systematic reviews, only data from primary literature meeting the inclusion criteria of the present work were extracted. The primary data were also examined individually to determine whether they described endpoints that may have been neglected in the review.

#### Study design

Included were randomised controlled trials (RCTs) from the primary search and those found on the basis of systematic reviews, meta-analyses or guidelines with a systematic search.

#### Participants

Included patients underwent systemic chemotherapy, surgery or irradiation, respectively, a simultaneous radio-chemotherapy. Patients were characterized by type and stage of cancer, age and sex.

#### Intervention

Studies were eligible if they conducted any systemic zinc treatment to cancer patients. Type of treatment, frequency and duration was extracted.

#### Comparison

Any kind of comparison was eligible in this review. This includes standard care, observation and placebo.

#### Outcomes

Primary endpoints included patient-centred symptoms or toxicities, e.g. mucositis, dry mouth, altered taste, oral pain, any other type of mucosal inflammation and individual aspects of nutrition. Secondary endpoints were survival data as overall survival, progression-free-, metastases-free- and disease-free survival, quality of life, fatigue, immune response and the toxicity of zinc.

## Results

**Fig. 1:**
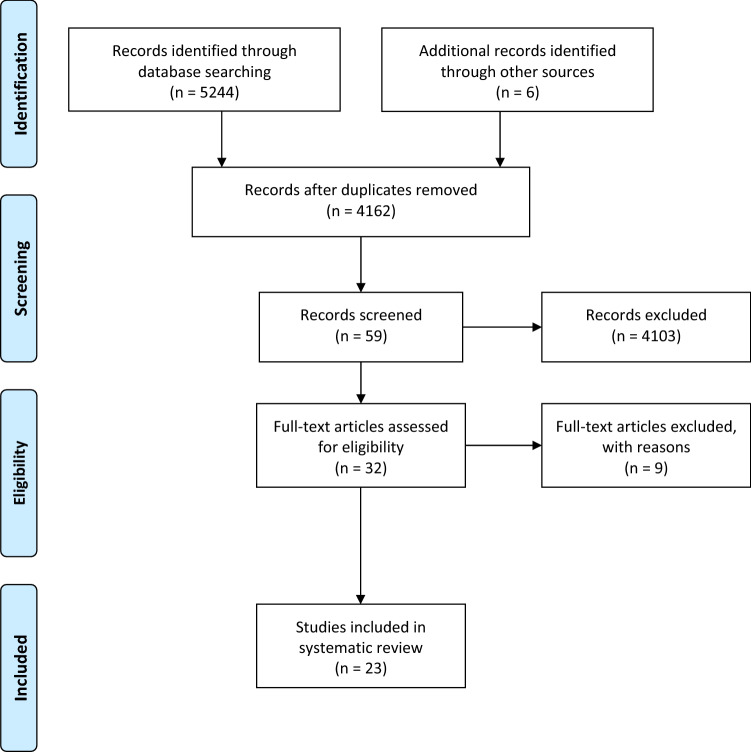
Flowchart

The systematic research revealed 5244 hits. Six studies were added by hand search. At first, duplicates were removed leaving 4162 studies. After screening titles and abstracts, 59 studies remained and underwent further investigation. Finally, 19 publications were included into our systematic review, including 1 meta-analysis and 18 RCT. In the meta-analysis, 5 studies were included of which all were considered relevant due to their reference to chemotherapy-induced side effects. Screening the reference lists of the studies and systematic reviews included after the first title–abstract screening, we found another 6 studies meeting the inclusion criteria. As a result, we included 19 publications reporting data from 23 relevant studies. Detailed characterization of the included studies may be seen in Table 3. The flow of studies through the review can be seen in Fig. 1.

**Table 3:** Characterization of the included studies

References	Endpoints	Outcomes
Tian et al. [15]	1. Incidence of mucositis 2. Severity of mucositis 3. Oral pain 4. Onset of mucositis 5. Toxicity 6. QoL	1. No significant differences between the groups Gholizadeh et al. [19], Mansouri et al. [18], Rambod et al. [16]: RR = 0.52, 95% CI 0.17–1.64, *p* = 0.27, *I*^2^ = 92% 2. No significant differences between the groups moderate/heavy severity: Gholizadeh et al. [19], Mansouri et al. [18]: RR = 0.62, 95% CI 0.11–3.56, *p* = 0.60, *I*^2^ = 65% heavy severity: Gholizadeh et al. [19], Mansouri et al. [18], Arbabi-Kalati et al. [20]: RR = 0.70, 95% CI 0.29–1.71, *p* = 0.44, *I*^2^ = 0% Mehdipour et al. [17]: tendency of lower moderate severity in arm A (in two of four weeks significant, *p* = 0.025) 3. Arbabi-Kalati et al. [20]: less pain in arm A (week 3–10, *p* < 0.005) Gholizadeh et al. [19]: no differences in intensity, greater efficacy in relieving pain in arm B (at the end of week 4, *p* = 0.03) 4. Rambod et al. [16]: no significant differences (*p* = 0.34) 5. Mansouri et al. [18]: no side effects 6. Arbabi-Kalati et al. [20]: no significant differences between the groups (*p* = 0.15–0.91)
Arbabi-Kalati et al. [20]	1. Mucositis 2. Xerostomia 3. Pain 4. QoL	1. Significant differences in week 8, 12, 16 and 20 concerning severity Week 8: arm A: mean (95% CI) = 1.54 (1.29–1.79), arm B: 2.2 (1.99–2.4) Week 20: arm A: 1.16 (0.57–1.17), arm B: 2.33 (0.89–3.76), *p* < 0.005 No significant differences concerning duration (*p* = 0.13) 2. Significant differences from week 4 Week 4: arm A: mean (95% CI) = 2.44 (2.19–2.68), arm B: 3.32 (3.09–3.54), *p* < 0.005 Intensity remained lower Week 20: arm A: mean (95% CI) = 1.16 (0.73–1.59), arm B: 2.5 (2.05–2.94), *p* = 0.0049 No significant differences in duration of necessary treatment (*p* = 0.23) 3. Significant differences from 6 to 20th week Week 6: arm A: mean (95% CI) = 5.56 (5.097–6.02), arm B: 7.48 (7.04–7.91), *p* = 0.003 Week 20: arm A: 4.00 (3.12–4.87), arm B: 7.00 (6.40–7.59), *p* = 0.0049 4. No significant differences
Braga et al. [36]	1. Antibody concentrations against serotypes 1, 5, 6B, 9 V, 14, and 18C 2. Seroconversion 3. Zinc plasma concentrations	1. Higher antibody concentration against all polysaccharides in both arms before and 4 weeks after vaccination *p* < 0.01 16 weeks after vaccination significant higher concentrations of PS6- specific antibodies in arm A Arm A: mean (95% CI) = 2.96 (1.74–5.03), arm B: mean (95% CI) = 10.75 (5.37–21.54), *p* < 0.01 2. No significant differences 3. Higher zinc plasma concentration after zinc intake Arm A: before vaccination: mean (SD) = 86.0 (14.1), after 16 weeks: mean (SD) = 128.9 (33.4), *p* = 0.01 After 16 weeks: arm B: mean (SD) = 89.2 (19.0), *p* = 0.001
Ertekin et al. [26]	1. Oral mucositis: duration, severity, onset 2. Weight	1. Significant differences in onset Arm A: median (range) week 3 (0–5), arm B: week 2 (2–3), *p* < 0.05 Significant differences in severity Arm A: median (range): 1 (0–2), arm B: 3 (2–3), *p* < 0.05 Significant differences in RT dose leading to mucositis Arm A: median (range): 3600 (2400–4400), arm B: 2000 (1800–2800), *p* < 0.01 6 weeks after RT, mucositis less frequently in arm A Arm A: 6.7%, arm B: 83.3%, *p* < 0.01 2. No significant differences (*p* = no information)
Gorgu et al. [23]	1. Oral mucositis 2. Esophagitis 3. Serum zinc level	1. No significant differences Grade 0, 1, 2, 3 in arm A: 12, 7, 5, 0, in arm B: 3, 6, 6, 1, *X*^2^ = 5.174, *p* = 0.159 2. No significant differences Grade 0, 1, 2, 3 in arm A: 6, 10, 6, 2 in arm B: 2, 6, 7, 1, *p* = 0.159 3. After the treatment in arm B significant lower Mean: no information, *p* = 0.05
Halyard et al. [31]	1. Onset of taste alteration 2. Incidence of taste alteration 3. QoL 4. Toxicity 5. Weight	1. No significant differences Arm A: median interval = 2.3 weeks, arm B: median interval = 1.6 weeks, *p* = 0.09 2. No significant differences Arm A: 73%, arm B: 84%, *p* = 0.16 3. No significant differences 4. More often moderate or severe dysphagia in arm A; otherwise rare with comparable frequencies and severity dysphagia: arm A: 7%, arm B: 4%, *p* = 0.02 5. Better maintenance of weight in arm A Arm A: 99%, Arm B: 92%, *p* = 0.04
Iovino et al. [37]	1. Toxicity	1. No significant differences
Lin et al. [25]	1. Grade 2 and 3 mucositis 2. Grade 2 and 3 dermatitis 3. Toxicity	1. Significant earlier appearance in arm B Grade 2: *p* = 0.017, grade 3: *p* = 0.0003 Less severity in Arm A, but 2 weeks after RT similar improvement *p* = 0.003 2. Significant earlier appearance in arm B Grade 2: *p* = 0.014, grade 3: *p* = 0.0092 Less severity in Arm A, but 2 weeks after RT similar improvement *p* = 0.003 3. No side effects
Lin et al. [34]	1. OS 2. LFS 3. MFS 4. DFS	1. No significant differences Hazard ratio (95% CI) = no information, *p* = 0.19 2. Tendency towards shorter LFS in arm B but not significant Hazard ratio (95% CI) = 1.64 (0.92–2.93), *p* = 0.092 Subgroup: stage III–IV cancer with concurrent chemotherapy treatment: significantly poorer LFS in arm B Hazard ratio (95% CI) = 3.01 (1.1–8.23), *p* = 0.032 3. No significant differences *p* = 0.35 4. No significant differences *p* = 0.54
Lin et al. [34]	1. OS 2. DFS 3. LFS 4. MFS	1. Significant better in arm A Death: arm A: *n* = 5 (29%), arm B: *n* = 11 (65%), *p* = 0.044 2. Significant better in arm A Recurrence: arm A: *n* = 7 (41%), arm B: *n* = 13 (76%), *p* = 0.033 3. Significant better in arm A Progression: arm A: *n* = 3 (18%), arm B: *n* = 10 (59%), *p* = 0.007 4. No significant differences Occurrence arm A: *n* = 6 (35%), arm B: *n* = 9 (53%), *p* = no information
Lin et al. [24]	1. Grade 2 und 3 mucositis	1. Earlier appearance in arm B Grade 2: *p* = 0.009, grade 3: *p* = 0.001 Shorter average duration in arm A Arm A: 3.55 weeks, arm B: 4.46 weeks, *p* = 0.033 Subgroup oral carcinoma: Earlier appearance in arm B (*p* < 0.001) Shorter average duration in arm A Arm A: 3.12 weeks, arm B: 5.14 weeks, *p* = 0.001 Subgroup nasopharyngeal carcinoma: No significant differences in onset and duration Arm A: 3.68 weeks, arm B: 4.10 weeks, *p* = 0.462
Lyckholm et al. [30]	1. Changes in taste and smell	1. No significant differences: trend toward improvement over time in all groups but non-significant worsening in loss of smell in the zinc group
Moslemi et al. [27]	1. Mucositis	1. Highest severity in arm B (*p* < 0.0001) Significant differences in OMAS value (*p* < 0.05) Significant differences in the appearance in the first week prevalence arm A: 40%, arm B: 70.5%, *p* < 0.0001 Lower severity in arm A in week 2–7 and 8 (*p* < 0.003)
Najafizade et al. [29]	1. Detection and recognition of the four taste qualities bitter, sour, sweet and salty	1. Significant worsening in all four qualities in arm B at the end of RT (p’s ≤ 0.03) In arm A only change for sour (*p* = 0.038) Significant worsening in all four qualities in arm B 1 month after RT (*p* = 0.001) In arm A only higher threshold for perception of salty (*p* = 0.046) No group comparisons
Ribeiro et al. [32]	1. Fatigue 2. QoL 3. BMI	1. No significant differences 2. No significant differences 3. No significant differences Baseline: arm A: mean (SD) = 24.8 (5.9), arm B: 24.9 (5.1) 4th cycle of CTX: arm A: 23.9 (5.1), arm B: 24.2 (6.5), *p* = ns
Ripamonti et al. [28]	1. Taste acuity: detection and recognition 2. Toxicity	1. More deterioration in taste accuracy in arm B during RT Faster regeneration of taste accuracy in arm A one month after RT Significant differences in the perception of bitter and the detection of salty in arm A during RT Bitter: *p* = 0.015, salty: *p* = 0.001 Significant differences in the detection of salty, sweet and sour in arm A after RT Salty: *p* = 0.0241, sweet: *p* = 0.019, sour: *p* = 0.028 2. No significant differences
Sangthawan et al. [22]	1. Development of oral mucositis und pharyngitis ≥ 2nd grades 2. Oral and throat pain 3. Toxicity 4. Weight	1. No significant differences Grade 2: *p* = no information, grade 3: mucositis: *p* = 0.54, pharyngitis: *p* = 0.84 No differences in mean radiation doses until onset mucositis: *p* = 0.96, pharyngitis: *p* = 0.59 2. No significant differences (*p* = 0.77) No significant differences in using analgesics (*p* = 0.71) 3. Nausea and vomiting (in most cases mild, 1 patient from arm A with moderate severity) 4. No significant differences (*p* = no information)
Sangthawan et al. [33]	1. OS 2. PFS 3. Toxicity	1. No significant differences (*p* = 0.55) 2. No significant differences (*p* = 0.39) 3. No significant differences (*p* = 0.67)
Watanabe et al. [21]	1. Oral mucositis 2. Pain 3. Xerostomia 4. Taste disturbance 5. Use of analgesics 6. Oral intake 7. Amount of daily meals	1. Significant differences in occurrence of grade ≥ 2 Arm A: 40%, arm B: 86.7%, *p* = 0.009 2. Significant differences in grade ≥ 2 Arm A: 33.3%, arm B: 86.7%, *p* = 0.003 3. Significant differences in occurrence of grade ≥ 2 Arm A: 13.3%, arm B: 73.3%, *p* = 0.001 4. Significant differences Arm A: 19%, arm B: 87%, *p* = 0.0002 5. Reduced use of analgesics (*p* = 0.0025) 6. No significant differences Arm A: 40%, arm B: 12.5%, *p* = 0.113 7. Significant larger amount of meals in arm A Arm A: 78.8 (± 31.2%), arm B: 30.7 (± 37.9%), *p* = 0.002

*CTX* chemotherapy, *DFS* disease-free survival, *LFS* local-free survival, *MFS* metastases-free survival, *ns* not significant, *OMAS* Oral Mucositis Assessment Scale, *OS* overall survival, *QoL* quality of life

Besides other topics or study designs, 6 systematic reviews were excluded due to methodical issues or small size. Furthermore, 3 RCTs with multiple interventions were excluded as the effects of the single parts of these interventions were not analysed separately. A list of the excluded studies combined with the reason for their exclusion can be found in Table 4.

**Table 4:** Excluded studies

References	Study type	Reason for exclusion
Bumrungpert et al. [38]	RCT	Intervention with a supplement containing a combination of zinc and selenium (zinc 2.64 mg/day and selenium 0.76 mg/day)
Chan et al. [39]	SR	Included only one study related to zinc (Lin [25])
Federico et al. [40]	RCT	Intervention with a supplement containing a combination of zinc and selenium (selenium 200 μg/day and zinc 21 mg/day)
Lee [41]	SR	No separate evaluation of the zinc studies (meta-analyses for all minerals together)
Posadzki et al. [42]	SR	Included only one study related to zinc (Schröder [43]) Intervention with a combination of supplements
Schröder et al. [43]	RCT	Intervention with a combination of supplements (soy, isoflavones, lycopene, silymarin and antioxidants as main ingredients)
Thomsen [44]	SR	Not enough study details: no evaluation of risk of bias, no reports of study sample sizes
Wong et al. [45}	SR	Included only one study related to zinc (Lin [25])
Yasueda [46]	SR	No evaluation of the risk of bias of the included studies

### Patient’s characteristics of included studies

Concerning all relevant studies, 1180 patients were assessed. Due to drop-outs, only 1120 of 1180 patients were included into our systematic review. The mean age of patients in the individual studies was 29–63 years with a range over all studies from 18 to 88 years. 420 participants were female and 760 males. The patients suffered from head and neck cancer (*n* = 667), leukaemia or lymphoma (*n* = 340), colorectal cancer (*n* = 51), other gastrointestinal cancers (*n* = 4), lung (*n* = 16), breast (*n* = 31) or prostate cancer (*n* = 8) or other cancer types (*n* = 3).

### Risk of bias in included studies

The methodical quality was assessed with SIGN checklist for controlled trials Version 2.0 [11]. The results are presented in Table 5. 13 of the included studies have moderate, 3 have high, and 2 studies are of poor quality.

**Table 5:**
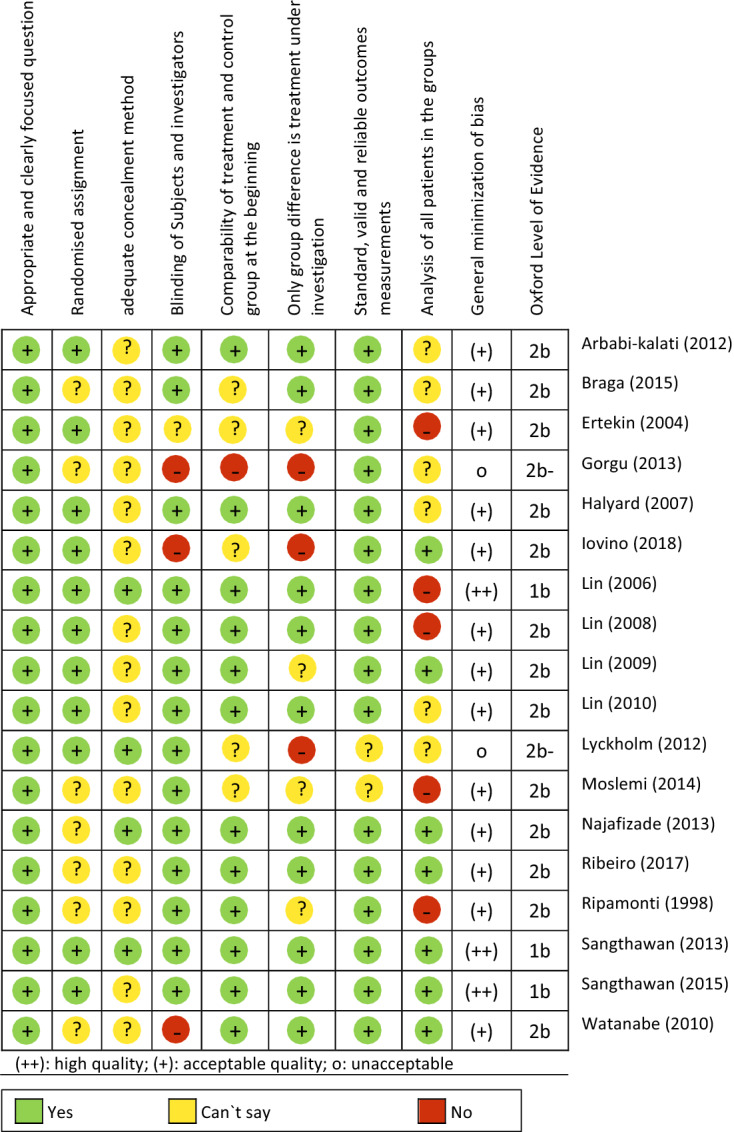
Methodical quality of the included RCTs

### Primary endpoints: efficacy of complementary zinc therapy

#### Chemotherapy-induced mucositis

Tian et al. [15] carried out a systematic search on three databases and included five RCTs, which together included 352 patients (per trial: 30–140, median: 60) and were all double-blinded. The indication of the patients was not described in detail; however, it was a prerequisite that the patients were on chemotherapy treatment and received either zinc sulphate or an identical looking and tasting placebo in the examined arms. The effect of zinc intake on the occurrence, onset and severity of oral mucositis was calculated in terms of a meta-analysis. Analyses of three RCTs showed no significant differences in the occurrence of oral mucositis between intervention and control arms (RR = 0.52, 95% CI 0.17–1.64, *p* = 0.27, *I*^2^ = 92% [16–19]), just as little as in their severity. Calculations based on two RCTs showed no difference between the arms in terms of moderate and severe mucositis (RR = 0.62, 95% CI 0.11–3.56; *p* = 0.60; *I*^2^ = 65% [18, 19]) and also regarding the severe form of mucositis, the results of the analyses based on three RCTs were not significant (RR = 0.70, 95% CI 0.29–1.71, *p* = 0.44, *I*^2^ = 0% [18–20]). Data on the onset of mucositis did not allow analysis. Results from Rambod et al. [16] concluded that there were no significant differences concerning this endpoint either (t = − 0.95, *p* = 0.34).

In summary, based on the five studies of this meta-analysis [15], there are *no significant effects of zinc intake on* the occurrence, onset or severity of *oral mucositis due to chemotherapy.*

#### Radiotherapy-induced mucositis

Seven further RCTs, dealt with the effect of zinc on oral mucositis as an acute toxicity of irradiation or chemoradiotherapy [21–27]. Five of them found significant results. The patients examined here had all a diagnosis of head and neck carcinoma and were treated with radiotherapy (RT) and in some cases with chemoradiation (RC). In the study by Ertekin et al. [26], 27 patients were included and examined over a period of up to 13 weeks. Endpoints were onset, duration and severity of oral mucositis. The authors concluded that mucositis started later in the zinc arm (zinc arm: median: week 3, placebo arm: week 2, *p* < 0.05), developed with a higher radiation dose (zinc arm: median (range): 3600 cGy (2400–4400 cGy), placebo arm: 2000 cGy (1800–2800 cGy), *p* < 0.01), was less severe (zinc arm: median: grade 1 RTOG, placebo arm: grade 3 RTOG, *p* < 0.05) and lasted shorter than in the placebo arm (after 6 weeks: zinc arm: 6.7%, placebo arm: 83.3%, *p* < 0.01). Lin et al. [25] focused primarily on the more severe forms of 2nd and 3rd grade mucositis, comparing zinc with a placebo made from soybean oil. In 97 patients, they also examined the time of onset and the severity of oral mucositis and found significant differences between the arms. Mucositis occurred earlier in the placebo arm than in the zinc arm (grade 2 RTOG: *p* = 0.017, grade 3 RTOG: *p* = 0.0003) and was less severe in the latter than in the placebo arm (*p* = 0.003). After the end of radiotherapy, similar improvements were seen in both groups. A subsequent subgroup analysis of the study sample already described [25] included only patients with nasopharynx (*n* = 40) or oral cancer (*n* = 43). Lin et al. [24] replicate the results only for the subgroup of patients with oral cancer in which the mucositis in the zinc arm started later (*p* < 0.001) and lasted shorter (zinc arm: 3.12 weeks, placebo arm: 5.14 weeks, *p* = 0.001), but not for patients with nasopharyngeal carcinoma (duration: zinc arm: 3.68 weeks, placebo arm: 4.10 weeks, *p* = 0.462). Two other RCTs that found significant differences in favour of zinc were carried out by Moslemi et al. [27] and Watanabe et al. [21]. Moslemi et al. [27] recruited a sample of 37 people, showing that patients in the zinc arm were affected by mucositis less early (prevalence in week 1: zinc arm: 40%, placebo arm: 70.5%, *p* < 0.0001) and that it was less intense when it appeared compared to the placebo arm (*p* < 0.003). Watanabe et al. [21] supported the position that zinc can positively influence the occurrence of oral mucositis. They examined 31 patients over a period of 10 months and randomly gave the patients either a polabrezinc or azulene solution. They found that mucositis ≥ 2nd degree occurred significantly less frequently in the zinc arm than in the azulene arm (zinc arm: 40%, control arm: 86.7%, *p* = 0.009).

Among the RCTs found, there were two of which the analyses yielded no significant results. The first by Gorgu et al. [23] with a sample of 40 patients, of whom 16 received zinc for an unspecified duration, found no group differences in the occurrence of mucositis (grade 0, 1, 2, 3 RTOG in the zinc arm: *n* = 12, 7, 5, 0, in the control arm: *n* = 3, 6, 6, 1, X^2^ = 5,174, *p* = 0.159) compared to the other 24 who were only treated by radiotherapy, although the serum zinc level in the intervention arm was significantly higher after treatment than in the control arm (*p* = 0.05). Sangthawan et al. [22] did not find any significant differences in the occurrence of oral mucositis in any of the weeks during radiotherapy as well. Their study included 144 patients. Over a period of two months, in which half of the patients received zinc sulphate and the other a placebo, the severity of mucositis grade 3 between the arms was comparable (*p* = 0.54).

In total, five of the seven other RCTs show *effects of zinc on* the onset, severity and duration of *oral mucositis due to radiotherapy or radio-chemotherapy* (except in patients with nasopharyngeal carcinoma).

#### Oral pain

Regarding the studies on the influence of zinc on patients` oral pain, two out of three RCTs found significant differences. The study by Arbabi-Kalati et al. [20] was already included in the meta-analysis by Tian et al. [15] but examined additional endpoints. The sample included 50 patients with various cancer diagnoses under chemotherapy who were observed for up to 20 weeks in addition to their concurrent chemotherapy. The authors reported significant differences in pain between the arms from the 6th to the 20th week of the survey (week 6: zinc arm: mean (95% CI) = 5.56 (5.097–6.02), placebo arm: 7.48 (7.04–7.91), *p* = 0.003, week 20: zinc arm: 4.00 (3.12–4.87), placebo arm: 7.00 (6.40–7.59), *p* = 0.0049). Watanabe et al. [21] reported significant differences in pain ≥ 2nd degree in patients with RT or RC treatment (zinc arm: 33.3%, placebo arm: 86.7%, *p* = 0.003). In contrast, the calculations by Sangthawan et al. [22] rather concluded that zinc had no benefit on the perceived oral and throat pain of the patients who were also treated with radiotherapy here (no differences between the arms, oral pain: *p* = 0.77, throat pain: *p* = 0.47) and that there was no effect on use of analgesics (*p* = 0.71).

For patients undergoing radio- or radio-chemotherapy, there are two studies with ambiguous results regarding the effect of zinc on pain. One study shows *positive effects of zinc on pain* in chemotherapy patients.

#### Xerostomia

There are two RCTs concerning the occurrence of xerostomia and zinc administration [20, 21]. From the fourth week onwards, Arbabi-Kalati et al. [20] found significant differences in the intensity of the xerostomia between the arms (week 4: zinc arm: mean (95% CI) = 2.44 (2.19–2.68), placebo arm: 3.32 (3.09–3.54), *p* < 0.005). However, the treatment of these complaints took the same amount of time in both arms (*p* = 0.23). The study by Watanabe et al. [21] concluded that grade 2 xerostomia occurred significantly less frequently in the zinc arm than in the placebo arm (arm A: 13.3%, arm B: 73.3%, *p* = 0.001).

Both studies, therefore, show a *positive influence of zinc substitution on xerostomia*, on the one hand on the severity and on the other hand on the frequency. There was no effect on the duration.

#### Dysgeusia

Loss of taste could be the acute as well as late toxicity due to radiotherapy in the head and neck area. So, another commonly studied endpoint related to zinc was the occurrence of dysgeusia, which was analysed in five RCTs [21, 28–31]. Except in the Lyckholm et al. [30] study, patients with head and neck cancer who underwent radiotherapy and received either zinc or a placebo in randomly divided arms were examined. Halyard et al. [31] examined the onset and general occurrence of dysgeusia on the basis of 159 patients, whereby the calculations showed no differences between the arms, neither in the time interval until the first occurrence, nor in the time until recovery or in general occurrence (onset: zinc arm: median interval = 2.3 weeks, placebo arm: median interval = 1.6 weeks, *p* = 0.09; proportion of patients that reported recovery: zinc arm: 5%, placebo arm: 16%; general occurrence: zinc arm: 73%, placebo arm: 84%, *p* = 0.16). Like Halyard et al. [31], Lyckholm et al. [30] did not report any significant results. They analysed 41 patients with various cancer diagnoses and chemotherapy treatments who were given either zinc or a placebo for 3 months. The participating patients should assess the perceived changes in taste and smell themselves on a scale. On the basis of the non-significant results between the groups (changes/loss of sensory stimuli not significant, *p* = no information), the authors advise against treatment with zinc.

In contrast, three other RCTs found significant group differences in favour for zinc. Najafizade et al. [29] examined the perception and recognition of the four tastes qualities salty, sweet, sour and bitter in 35 patients. The placebo arm showed deterioration of all four flavour qualities by the end of radiotherapy (*p* ≤ 0.03 for all), whereas the zinc arm only showed a deterioration of the flavour or of sour taste (*p* = 0.038). Even one month after the therapy, the threshold for identifying the tastes had risen significantly for all four flavours in the placebo arm (*p* = 0.001). The zinc arm had only deteriorated in terms of salty (*p* = 0.046). However, these results only describe the courses within the groups and no intergroup comparison. Ripamonti et al. [28] used the same survey method in their study and investigated taste perception and recognition in 18 patients. During radiotherapy, the placebo arm deteriorated more than the zinc arm, and the latter recovered from the changes faster than the placebo arm within a month of treatment. With regard to the individual flavours, there were some significant differences in favour of the zinc arm with regard to perception (while RT: bitter: *p* = 0.015) and recognition (while RT: salty: *p* = 0.001, after RT: salty: *p* = 0.0241, sweet: *p* = 0.019, sour: *p* = 0.028). Watanabe et al. [21] also found significant differences in the occurrence of dysgeusia (zinc arm: 19%, azulene arm: 87%, *p* = 0.0002).

In summary, we have registered a common *trend to improving the taste* during radiotherapy if zinc is substituted. In contrast, chemotherapy-related dysgeusia is not improved by zinc.

#### Nutritional intake and weight

Watanabe et al. [21] also considered the restrictions on oral food intake and the number of daily eaten meals in more detail. The authors found no significant differences in the patients' ability to ingest food orally (zinc arm: 40%, placebo arm: 12.5%, *p* = 0.113), but the number of meals consumed daily differed significantly between the arms (zinc arm: 78.8 (± 31.2%), placebo arm: 30.7 (± 37.9%), *p* = 0.002).

Loss of weight was observed in four of the RCTs. Halyard et al. [31] showed that patients in the zinc arm were able to maintain their weight over the duration of the treatment easier than in the placebo arm (zinc arm: 99%, placebo arm 92%, *p* = 0.04). In the study by Sangthawan et al. [22], patients in both arms lost weight, but the results were not significant in none of the measurement time points (*p* = no information). Ribeiro et al. [32] included 24 patients with stages II to IV of colorectal adenocarcinoma in their study and examined the effects of zinc or a placebo over a period of approximately 16 weeks in which the patients were treated with chemotherapy. They assessed the BMI, whereby they could not find any significant differences from the baseline to the fourth cycle of chemotherapy (T0: zinc arm: mean (SD) = 24.8 (5.9), placebo arm: 24.9 (5.1), T4: zinc arm: 23.9 (5.1), placebo arm: 24.2 (6.5), *p* = not significant). Although the serum zinc levels of the two groups differed significantly before the 4th cycle (in favour of the intervention arm, *p* = no information). Ertekin et al. [26] reported the weight of the patients, which in their case did not differ between the arms (*p* = no information).

#### Dermatitis

Lin et al. [25] recorded mucositis and the effects of zinc on 2nd and 3rd degree dermatitis according the RTOG scale. There were significant differences in favour of zinc, since grade 2 and 3 dermatitis appeared earlier in the placebo arm than in the zinc arm (grade 2 RTOG: *p* = 0.014, grade 3 RTOG: *p* = 0.0092) and was more severe (*p* = 0.003). Two weeks after radiotherapy, both arms showed a similar improvement. Here, too, the authors reported that the zinc level of the intervention arm differed significantly from the placebo arm (*p* = 0.02).

### Secondary endpoints: efficacy of complementary zinc therapy

#### Survival

Overall survival and the time intervals until local or distant recurrence of the disease were examined by Sangthawan et al. [33], Lin et al. [34], Lin et al. [35] in RCTs. Sangthawan et al. [33] observed 72 patients with head and neck cancer during radiotherapy treatment and randomized them into a zinc or placebo group. They found no significant differences in the overall survival time associated with the administration of zinc (*p* = 0.55). The progression of the disease could not be stopped by zinc (*p* = 0.39). The two studies by Lin et al. [35] and Lin et al. [34] are both based on the sample of Lin et al. [25], so no new patients were collected for the analyses. Comparable to the results of Sangthawan et al. [22, 33], Lin et al. [35] also found no significant differences neither on overall survival (hazard ratio (95% CI) = no information, *p* = 0.19), the time to local tumor progression (hazard ratio (95% CI) = 1.64 (0.92–2.93), *p* = 0.092) or until the appearance of distant metastases (no information, *p* = 0.35), nor in the disease-free period (no information, *p* = 0.54). Only the local recurrence of the tumour was able to be delayed by zinc, especially for patients with stage III-IV cancer and concurrent chemotherapy. In the study by Lin et al. [34], the 34 patients with nasopharyngeal carcinoma in the III or IV stage were analysed separately from the sample, which originally comprised 97 patients. These calculations showed significant differences. After 68 months, patients in the zinc arm showed better outcomes in terms of overall survival (zinc arm: 29%, placebo arm: 65%, *p* = 0.044), disease-free survival (zinc arm: 41%, placebo arm: 76%, *p* = 0.033) and time to local recurrence compared to the placebo arm (zinc arm: 18%, placebo arm: *n* = 59%, *p* = 0.007). The occurrence of local metastases did not differ significantly between the arms (zinc arm: 35%, placebo arm: 53%, *p* = no information). All of the three studies summarized here examined how well zinc could be absorbed by the patients over the period of treatment. It was consistently shown that the serum zinc level in the intervention arms rose significantly (p`s < 0.05).

#### Quality of life

Quality of life parameter is summarizing the control of all symptoms during the cancer period. So, we have declared them as secondary endpoint in order to document this specific relation of dependence. The effects of zinc on patients` quality of life were examined in three RCTs [20, 31, 32], of which none found significant results. The analysis by Ribeiro et al. [32] revealed no significant differences between the arms (*p* = no information), although the zinc serum level in the intervention arm was significantly higher than in the placebo arm at the end of their examination (*p* = no information). The quality of life in the placebo arm deteriorated significantly over the course of the four chemotherapy cycles (T1: mean (SD) = 126 (16), T4 = 116 (27), *p* = 0.02). Accordingly, the authors discuss that zinc may protect against the worsening of quality of life in patients with colorectal cancer. The studies by Arbabi-Kalati et al. [20] and Halyard et al. [31] were unable to find any significant effects of zinc on quality of life.

#### Fatigue

In addition to quality of life and BMI, Ribeiro et al. [32] reported fatigue as an endpoint. To summarize, there was no difference between the arms through their intervention (*p* > 0.05).

#### Immune response after vaccination

The study by Braga et al. [36] dealt with the antibody concentration and seroconversion rate after pneumococcal vaccination. The 25 participating patients with colorectal cancer were given either zinc or a placebo for 16 weeks. The analyses showed that the antibody concentration increased in all patients (*p* < 0.01). At week 16, contrary to expectations, there was one significantly higher polysaccharide concentration (PS6) in the placebo arm compared to the zinc arm (zinc arm: mea* n* = 2.96, 95% CI: 1.74–5.03; placebo arm: mea* n* = 10.75, 95% CI: 5.37–21.54, *p* < 0.01). Regarding the seroconversion rate, there were no significant differences between the arms. At the same time, the analyses showed that the serum zinc level in the intervention arm was significantly higher after 16 weeks than in the placebo arm (*p* = 0.001).

#### Adverse events

A systematic assessment of toxicity of zinc was done in six RCTs [22, 25, 28, 31, 33, 37]. Halyard et al. [31] administered 45 mg of zinc sulphate three times a day to 76 of 159 patients. The intervention lasted over the period of the radiotherapy treatment until one month after its completion. They noticed a more frequent occurrence of moderate and severe dysphagia in the zinc arm compared to the placebo arm (zinc arm: 7%, placebo arm: 4%, *p* = 0.02), but no other significant differences in frequency and severity of side effects were found. Sangthawan et al. [22] administered zinc sulphate to 72 of 144 patients, parallel to their radiotherapy treatment, three times a day in a higher dose of 50 mg. The treatment lasted approximately 3 months and the authors reported that side effects such as nausea and vomiting were mostly mild. A patient in the intervention arm had shown moderate manifestations of nausea and vomiting. No group comparison was calculated, but no events of pain, fever, diarrhoea or fatigue were reported in either groups. No additional side effect was reported for the interventional group. The four other RCTs did also not notice any significant group differences in the occurrence of side effects due to zinc. Lin et al. [25] gave their patients a comparably smaller amount of zinc with 25 mg three times a day and examined the 97 patients (49 of them in the intervention arm) over a period of two months. According to their results, no side effects or interactions were observed. Ripamonti et al. [28] set a higher dose and administered 45 mg three times a day to their patients, with their study extending over three months. They also reported that no side effects occurred. In the study by Sangthawan et al. [33], the dose of zinc was 50 mg three times a day, given over the duration of the radiotherapy. Although the authors reported vomiting as side effect, there was no significant group difference (*p* = 0.67). With 600 mg daily over a period of 95 days, the dose of zinc administration was the highest in the study by Iovino et al. [37]. The results showed that 55.5% of the patients had side effects. There were cases of nausea and diarrhoea and one patient per arm had fever > 38 °C. However, side effects did not differ significantly between groups (*p* = 1.).

In other studies, side effects were reported but without a systematic assessment. Ertekin et al. [26] noted the appearance of vomiting and nausea in 20% of the zinc group cases. The patients had received 50 mg zinc three times a day for 13 weeks. Lyckholm et al. [30] observed side effects such as diarrhoea, abdominal pain, cramps and diaphoresis, which they also assumed to be one of the reasons for the patient's early termination of the study. The investigations by Moslemi et al. [27], Najafizade et al. [29] and the study by Mansouri et al. [18] from the meta-analysis by Tian et al. [15] stated, that they did not find any side effects in their patients from taking zinc.

## Discussion

### Oral mucositis due to chemotherapy

In their meta-analysis, Tian et al. [15] reported the impact of zinc substitution on chemotherapy-induced inflammation of oral and oropharyngeal mucosa. The calculations were carried out on the base of very limited and sometimes very heterogeneous of data, which is particularly evident in the analyses of the occurrence of oral mucositis. Based on these data, zinc does not protect against chemotherapy-induced oral mucositis.

### Oral mucositis due to irradiation

Two studies on mucositis due to irradiation found no significant effect of zinc [15, 22, 23]. The study of Sangthawan et al. [22] was methodically well-conducted. In the study of Gorgu et al. [23], the main criticism is the unspecific differentiation between oropharyngeal mucositis and esophagitis which normally not should occur due to irradiation of the head and neck cancer. The sample size did not meet the requirements for the calculated analyses, which made the results more likely to be error-prone. Additionally, the two study groups differed in their characteristics right from the start and there was no information on the duration of the intervention and the randomization procedure.

On the other hand, five RCTs reported significant results concerning zinc and mucositis due to irradiation [21, 24–27]. Also, these studies have some risk of bias, such as the small sample size [21, 24, 26, 27] and the lack of proof of comparability of the groups at the beginning of the intervention [26, 27]

In summary, the results of 5/7 studies indicate that zinc is able to reduce mucositis during radio- or simultaneous radio-chemotherapy due to head and neck cancer. Overall, more RCTs indicate a positive effect of zinc, that is mucositis started later, turned out to be less strong and lasted shorter when zinc was supplemented.

Arbabi-Kalati et al. [20] and Watanabe et al. [21] also reported significant differences in the effect of zinc on patients` oral pain. Yet, unexplained attrition [20] and open design [21] leave uncertainties about the results. Sangthawan et al. [22], however, found no significant effects.

### Xerostomia

There are two studies that agreed that zinc could have a positive effect on xerostomia [20, 21]. The study by Arbabi-Kalati et al. [20] gives the impression that the patients were examined over 20 weeks, however, based on the information, it cannot be said whether all patients were actually evaluated, since no intention to treat analysis was planned and there was no information on drop-outs or attrition.

Overall, these two studies speak for the positive effect of zinc on xerostomia, but both should be viewed with caution due to methodologically drawbacks. Nevertheless, the studies could be a starting point for further research. Less saliva is one of the key points in the pathogenesis of oral and oropharyngeal inflammation, e.g. further research should try to explain how zinc is working in salivary gland tissue.

### Loss of taste (dysgeusia)

Loss of taste is a common side effect that occurs after 3 or 4 weeks of irradiation in the head and neck region. It is the result of changing salivary flow, inflamed mucosa and direct down-regulated sensitivity of taste receptors. The impact of zinc on dysgeusia has been studied in five studies, two of which showed no significant [30, 31] and three of which significant effects [21, 28, 29]. However, all studies also had methodological limitations. In the study by Halyard et al. [31], it remains unclear why only 49% of the patients carried out the complete study. Methodological limitations in the study by Lyckholm et al. [30] were among others a lack in the comparability of the groups and a high drop out with lack of comprehensive statistical reporting.

There was no intergroup comparison in the study by Najafizade et al. [29]. Meaningful results on differences in taste changes between the groups are, therefore, not available and the available data can at most be regarded as an indication of a positive effect of zinc. Ripamonti et al. [28] carried out a complex statistical analysis that showed significant results concerning zinc and individual flavours. The analysis was adapted to the size of the sample and included baseline values. Together with the study by Watanabe et al. [21], these three studies agree in their judgement on zinc and dysgeusia, but all three make their conclusions on a relatively small sample, which limits the generalizability and meaningfulness of the results.

All in all, the studies described here on dysgeusia have different methodological shortcomings. The trend indicates a positive effect of zinc on taste changes in cancer patients.

### Nutritional intake and weight

Oral mucosa, mild improvement of taste and dry mouth could lead to better nutritional intake and weight. Watanabe et al. [21] have described higher consumption of food if the patient received zinc substitution. Nevertheless, the authors concluded that zinc does not appear to have sufficient influence on the possibility of oral food intake.

The majority of the studies, therefore, suggests that zinc does not have a positive influence on the weight or food intake of the patients [22, 26, 31, 32].

### Dermatitis

In the high-quality study by Lin et al. [25], positive effects of zinc supplementation on dermatitis were found: dermatitis appeared earlier and was more severe in the placebo arm. Only patients with head and neck cancer were included and further studies are needed to check replicability and possible generalization. The reduction of skin inflammation could be seen as a model correlating to the discussion about mucositis above.

### Survival

Since the study by Sangthawan et al. [33] was methodologically well done, it can be concluded that zinc does neither have a positive influence nor harms the patient (see also adverse events). The results of the study by Lin et al. [35] can only be generalized to a very limited extent, since many analyses were calculated for very specific subgroups. The extent to which they were comparable and the size they comprised remains unclear. Concerning the study by Lin et al. [34], similar methodological deficits have to be stated.

Overall, the data from the studies of larger samples rather speak against an effect of zinc on overall or disease-free survival. However, this might vary for special subgroups, like for patients with concurrent chemotherapy or nasopharyngeal carcinoma in the III or IV stage.

### Quality of life and fatigue

None of the three RCTs that examined patients' quality of life found significant results [20, 31, 32]. The positive changes of taste, pain, and oral discomfort did make no impact on QoL.

Moreover, the study by Ribeiro et al. [32] assessed the effects of zinc on fatigue and found no differences between the study arms.

### Adverse events

The systematic study of side effects did not reveal any significant differences between the groups in the majority of the studies [22, 25, 28, 33, 37]. Only Halyard et al. [31] reported a more frequent occurrence of moderate and severe dysphagia in the zinc arm, but they gave no information on the exact duration of the intervention until the side effects occurred. Furthermore, the results of this study are contradicting regarding dysphagia and body weight.

All in all – also taking together the studies that reported side effects without systematic assessment – only a few adverse events are described with respect to zinc, which seem to be rather mild and are equal to symptoms in the control group even with a higher dose of zinc.

### Limitations of this work

There are some limitations of this systematic review which have to be mentioned. First, we excluded studies concerning children or teenager and only analysed studies with adult patients. Second, only studies in English or German language were included. This means that the search for zinc in connection with the treatment of cancer can still be expanded in further research.

Besides, we did not conduct a meta-analysis. The main reason for this was the great heterogeneity of the included studies. In fact, they examined very different cancer types, interventions and endpoints. In addition, the subgroups were small. Moreover, there are only small studies with more or less high risk of bias. Only one radio-protection study provided a statistical planning and had a sufficient number of participants. Since the quality of a meta-analysis would have suffered under the points mentioned, it was decided to summarize the studies in review form.

## Conclusion

Zinc substitution is able to help in protection against radiotherapy-induced inflammation of oral and oropharyngeal mucosa. No improvement is given in cases of mucositis due to chemotherapy. We have registered trends to less loss of taste and dry mouth during radiotherapy as well as mucositis-related oral pain. Zinc has no impact on the survival of tumour patients, e.g. it will not decrease the effect of basis anti-cancer therapy. For further research, a stringent planning of high-quality RCT’s with adequate numbers of participants and a comprehensive reporting of outcomes is needed.
